# Distribution of Major Health Risks: Findings from the Global Burden of Disease Study

**DOI:** 10.1371/journal.pmed.0010027

**Published:** 2004-10-19

**Authors:** Anthony Rodgers, Majid Ezzati, Stephen Vander Hoorn, Alan D Lopez, Ruey-Bin Lin, Christopher J. L Murray, Group Comparative Risk Assessment Collaborating

**Affiliations:** **1**Clinical Trials Research Unit, School of Population Health, University of AucklandNew Zealand; **2**Harvard School of Public Health, BostonMassachusettsUnited States of America; **3**School of Population Health, University of QueenslandBrisbaneAustralia; University of California at San FranciscoUnited States of America

## Abstract

**Background:**

Most analyses of risks to health focus on the total burden of their aggregate effects. The distribution of risk-factor-attributable disease burden, for example by age or exposure level, can inform the selection and targeting of specific interventions and programs, and increase cost-effectiveness.

**Methods and Findings:**

For 26 selected risk factors, expert working groups conducted comprehensive reviews of data on risk-factor exposure and hazard for 14 epidemiological subregions of the world, by age and sex. Age-sex-subregion-population attributable fractions were estimated and applied to the mortality and burden of disease estimates from the World Health Organization Global Burden of Disease database. Where possible, exposure levels were assessed as continuous measures, or as multiple categories. The proportion of risk-factor-attributable burden in different population subgroups, defined by age, sex, and exposure level, was estimated. For major cardiovascular risk factors (blood pressure, cholesterol, tobacco use, fruit and vegetable intake, body mass index, and physical inactivity) 43%–61% of attributable disease burden occurred between the ages of 15 and 59 y, and 87% of alcohol-attributable burden occurred in this age group. Most of the disease burden for continuous risks occurred in those with only moderately raised levels, not among those with levels above commonly used cut-points, such as those with hypertension or obesity. Of all disease burden attributable to being underweight during childhood, 55% occurred among children 1–3 standard deviations below the reference population median, and the remainder occurred among severely malnourished children, who were three or more standard deviations below median.

**Conclusions:**

Many major global risks are widely spread in a population, rather than restricted to a minority. Population-based strategies that seek to shift the whole distribution of risk factors often have the potential to produce substantial reductions in disease burden.

## Abstract 2

Analysis of 26 major global health risk factors by age, sex, exposure level, and epidemiological subregion reveals that many of them are widely spread in a population rather than restricted to a minority

## Introduction

Reliable and comparable analysis of risks to health is an important component of evidence-based policies and programs for disease prevention [1,2]. An important feature of risk assessment, with implications for specific interventions as well as broad prevention policies, is the distribution of disease burden among population subgroups. These subgroups may be defined by demographic factors, such as age and sex, or by socioeconomic status. Subgroups can also be defined by the level of exposure to a risk factor, if exposures are defined in multiple categories or continuously.

Understanding the distribution of risk-factor burden is particularly important for targeting interventions. For example, the large number of road traffic accident injuries and deaths among young adult males may be largely associated with binge alcohol consumption by this group. Interventions that focus on this population subgroup and their specific drinking behaviors may be more effective or cost-effective than, for example, raising alcohol taxes, which would have a more diffuse impact on alcohol consumption. On the other hand, the majority of effects from risk factors such as blood pressure have been found to be among those at moderately elevated levels, motivating interventions beyond those intended for clinical hypertension [3,4,5].

The distributions of the health effects of risk-factor exposure by age and sex or by exposure level have been studied in specific cohorts and for specific risk factors [6,7,8]. Most notably Rose's seminal work stated that “a large number of people exposed to a small risk may generate many more cases than a small number exposed to high risk” [9]. Using the data from a global and regional assessment of multiple major risk factors, this paper reports the distribution by exposure levels, age, and sex of disease burden attributable to several major risk factors. The findings of this analysis confirm that Rose's observations have global relevance and also illustrate important new patterns on specific distributions of disease burden for multiple risks, in different age groups, and in populations at various stages of economic development.

## Methods

The methods and data sources for individual risk factors and for estimating population attributable fractions (PAFs) and disease burden attributable to them have been fully described elsewhere [1,2] and are summarized below. The contribution of a risk factor to disease or mortality relative to some alternative exposure scenario (i.e., PAF, defined as the proportional reduction in population disease or mortality that would occur if exposure to the risk factor were reduced to an alternative exposure scenario [10]) is given by the generalized “potential impact fraction”:



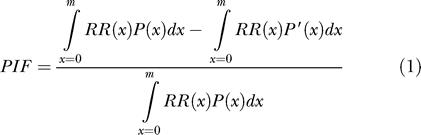



*RR*(*x*) is relative risk at exposure level *x, P*(*x*) is the population distribution of exposure, *P*′(*x*) is the alternative or counterfactual distribution of exposure, and *m* is the maximum exposure level.

The alternative (counterfactual) scenario used in this work is the exposure distribution that would result in the lowest population risk, referred to as the theoretical minimum-risk exposure distribution (Table 1) [1,2]. The theoretical minimum exposure distribution was zero in most cases since zero exposure reflected minimum risk (e.g., no smoking). For some risk factors, zero exposure was an inappropriate choice as the theoretical minimum, either because it is physiologically impossible (e.g., body mass index [BMI] and cholesterol) or because there are physical lower limits to exposure reduction (e.g., ambient particulate matter concentration and occupational noise). For these risk factors, the lowest levels observed in specific populations and epidemiological studies were used as the theoretical minimum. For factors with protective effects (i.e., fruit and vegetable intake and physical activity), a counterfactual exposure distribution was chosen based on levels in high-intake populations (e.g., fruit and vegetable intake in Greece) and the level to which the benefits may continue given current scientific evidence. Using theoretical minimum exposure distribution as the counterfactual has the advantage of providing a vision of potential gains in population health by risk reduction from all levels of suboptimal exposure in a consistent way across risk factors.

**Table 1:**
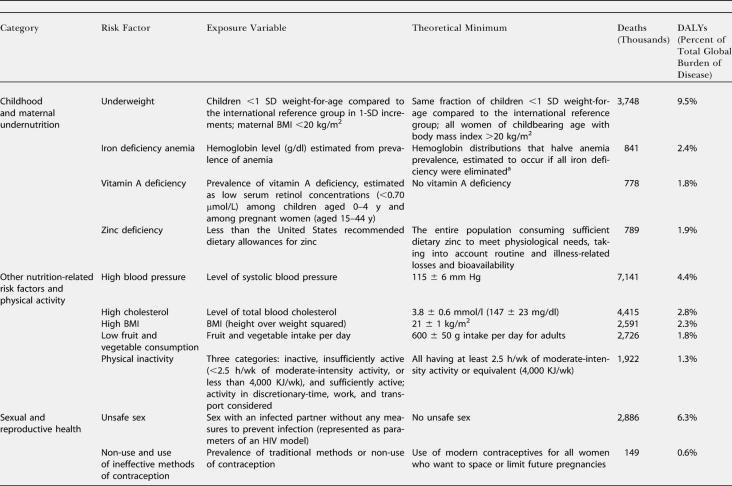
Leading Global Risk Factors, Exposure Variables, Theoretical Minima, and Attributable Deaths and Disease Burden (measured in DALYs) in 2000

See Table 1 in Ezzati et al. [2] for disease outcomes and data sources

^a^ The resulting hemoglobin levels vary across regions and age-sex groups (from 11.66 g/dl in children under five in a region of Southeast Asia to greater than 14.5 g/dl in adult males in developed countries) because the other risks for anemia (e.g., malaria) vary

^b^ Theoretical minimum for alcohol is zero, the global theoretical minimum. Specific subgroups may have a non-zero theoretical minimum

^c^ Theoretical minimum for lead is the blood lead levels expected at background exposure levels. Health effects were quantified for blood lead levels above 5 μg/dl where epidemiological studies have quantified hazards

**Table 1:**
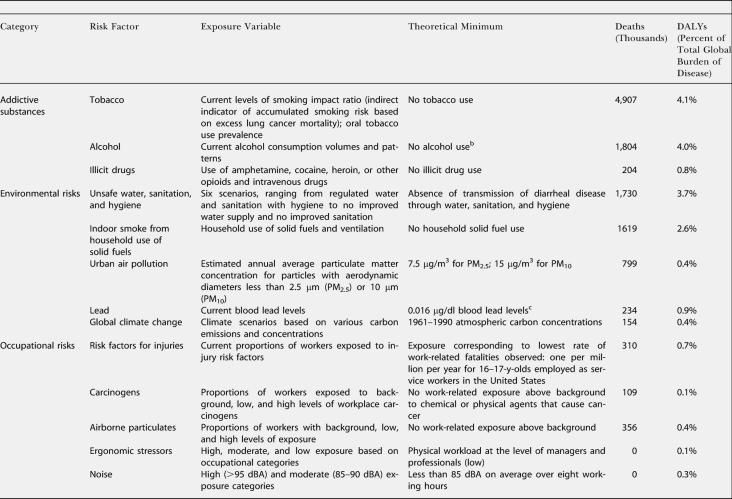
Continued

**Table 1:**
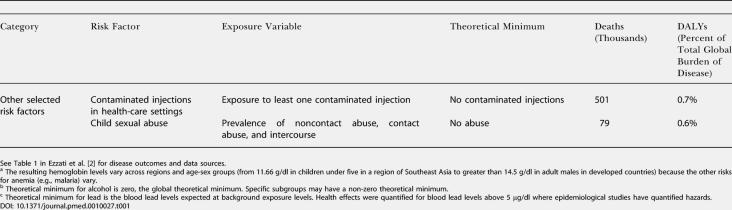
Continued

Estimates were made for eight age groups (0–4, 5–14, 15–24, 25–44, 45–59, 60–69, 70–79, and 80+ y), both sexes, and 14 Global Burden of Disease subregions (Table 2). PAFs were estimated for mortality and incidence and were applied to regional cause-specific mortality and disease burden from the World Health Organization (WHO) Global Burden of Disease database (Table 1). Burden of disease, reported annually in the annexes of the *World Health Report,* was expressed in disability-adjusted life-years (DALYs) with methods and assumptions described elsewhere [11]. Aggregate results (both mortality and disease burden) for all ages, sexes, and exposure levels have been reported elsewhere [1,2]. Many risks act simultaneously to cause disease, and joint effects have also been estimated [12], though the separate effects are presented in this paper. The aim of the analyses reported here was to obtain estimates of the distribution of disease burden by age, sex, and exposure level. To make separate estimates of disease burden by exposure level, the relationship in equation 1 was re-estimated with the entire exposure distribution divided into “narrow bands,” with each band corresponding to one level of exposure, and the estimation repeated for each such exposure band.

**Table 2:**
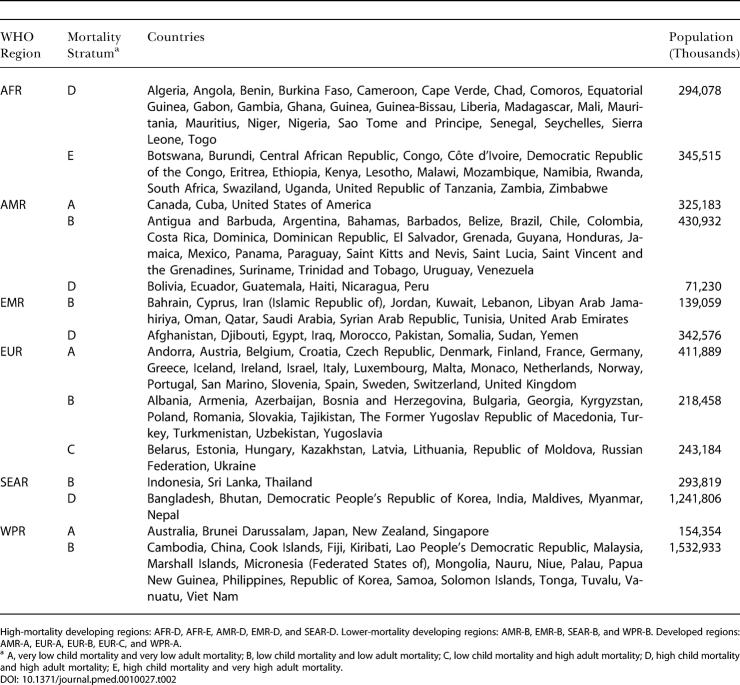
Global Burden of Disease 2000 Subregions

High-mortality developing regions: AFR-D, AFR-E, AMR-D, EMR-D, and SEAR-D. Lower-mortality developing regions: AMR-B, EMR-B, SEAR-B, and WPR-B. Developed regions: AMR-A, EUR-A, EUR-B, EUR-C, and WPR-A

^a^ A, very low child mortality and very low adult mortality; B, low child mortality and low adult mortality; C, low child mortality and high adult mortality; D, high child mortality and high adult mortality; E, high child mortality and very high adult mortality

## Results

The distribution of deaths and DALYs attributable to the risk factors by age and sex is shown in Table 3. Disease burden attributable to being underweight and to micronutrient deficiencies in children was equally distributed among males and females. The total all-age disease burden from iron and vitamin A deficiencies was greater in females because of effects on maternal mortality and morbidity conditions. The disease burden of other diet-related risks, physical inactivity, environmental risks, and unsafe sex (sex with an infected partner without any measures to prevent infection) occurred almost equally in males and females. However, approximately 80% of disease burden from addictive substances and 60%–90% from occupational risks occured among men (bearing in mind that the assessment considered only formal employment). Women experienced an estimated two-thirds of the disease burden from childhood sexual abuse and all of the burden caused by a lack of contraception.

**Table 3:**
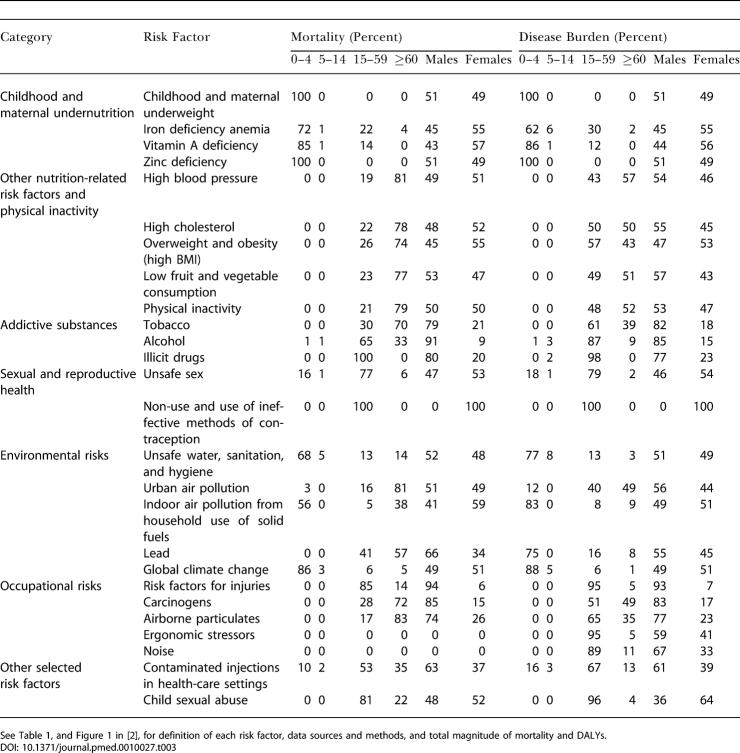
Distribution of Risk-Factor-Attributable Deaths and Disease Burden (DALYs) by Age and Sex

See Table 1, and Figure 1 in [2], for definition of each risk factor, data sources and methods, and total magnitude of mortality and DALYs

The estimated disease burden from childhood and maternal undernutrition; unsafe water, sanitation, and hygiene; and global climate change (much of whose estimated effects are mediated through nutritional and water variables) was almost exclusively among children under 5 y of age. For these risks, more than 85% of total attributable burden occurred in this age group, with the exception of iron deficiency, for which 30% of burden was borne by women of childbearing age. Only a small fraction of disease burden from the risk factors considered was among the 5–14 y olds. This was because some of the leading diseases of this age group (e.g., injuries and depression) have complex causes that could not easily be included in the current risk-based framework [12]. For other leading diseases of this group (e.g., diarrhea and lower respiratory infections), most epidemiological studies have focused on children under 5 y of age and do not provide hazard estimates for older children.

The disease burden from other diet-related risks, tobacco, and occupational risks (except injuries and back pain) was almost equally distributed among adults above and below the age of 60 y. For example, 43% of disease burden due to blood pressure and 61% of burden due to tobacco occurred in the 15–59-y age group. More than 90% of disease burden attributable to lack of contraception, illicit drugs, occupational ergonomic stressors, risk factors for injury, and childhood sexual abuse occurred in adults below the age of 60 y. Similarly, 67%–87% of the disease burden attributable to alcohol, unsafe sex, and unsafe health-care injections arose from events occurring between 15 and 59 y of age. Most of the risks whose hazards are concentrated in the younger adults are causes of injuries, neuropsychiatric diseases, maternal conditions, and HIV/AIDS. Assessment by the level of economic and demographic development illustrated that, with the exception of alcohol, which has global presence, the majority of disease burden from risks for mortality and disease among young adults is concentrated in developing countries (see Figure 1 in [2]). Stratification of economic and demographic development was also a determinant of the age distribution patterns for risk factors for chronic diseases, which occurred in younger age groups in developing countries than in developed regions. For example, in high-mortality developing regions, 69% of the tobacco burden occurred in people aged 15–59 y, whereas this share was 63% for lower-mortality developing countries and 55% for developed countries.

**Figure 1:**
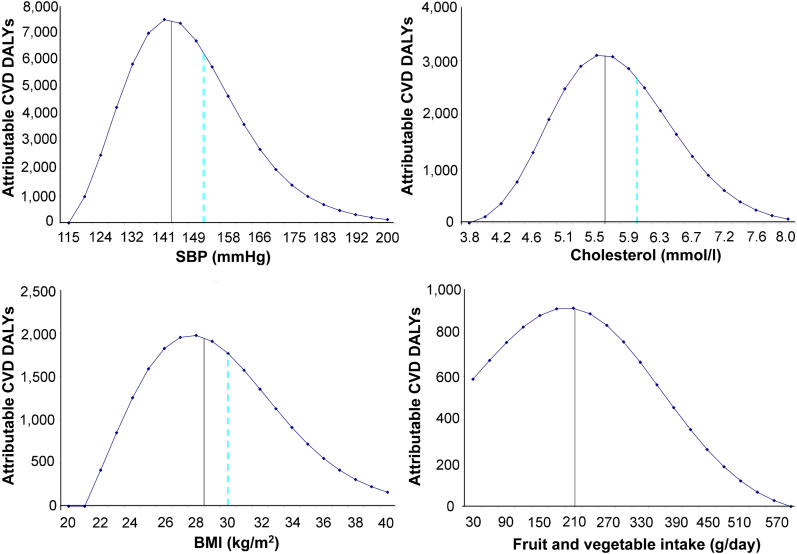
Distribution by Exposure Level of Attributable Disease Burden Due to Selected Continuous Risk Factors Figure 1 shows the distribution of the estimated cardiovascular disease (CVD) burden of disease (in DALYs) attributable to four major continuous risk factors, by exposure levels. Half the attributable burden occurs to the left of the solid vertical line and half occurs to the right. The dashed vertical lines indicate commonly used thresholds—150 mm Hg for hypertension, 6.0 mmol/l for hypercholesterolemia, and 30 kg/m^2^ for obesity. The blood pressure and cholesterol levels plotted are the estimated usual levels [22], which tend to have a smaller SD than levels based on one-off measurements commonly used in population surveys, because of normal day-to-day and week-to-week fluctuations. For example, the distribution of usual blood pressure is about half as wide as the distribution of one-off blood pressure measures, and so many fewer people would be classified as hypertensive (or hypotensive) if classifications were based on usual rather than one-off blood pressure. Thus, if a population mean SBP was 134 mm Hg, the SD of once-only measures might be 17 mm Hg (with about 18% of the population having one-off SBP over 150 mm Hg), and the SD of usual SBP based on many measures would be about 9 mm Hg (hence about 5% of the population would have usual SBP over 150 mm Hg).

The distributions of attributable risk-factor burden by exposure levels are shown in Table 4 for those risks quantified using categorical variables and in Figure 1 for those with continuous variables. For most of these risks a substantial proportion of attributable disease burden occurred among those with modest elevations of risk. For example, while 35% of the large disease burden from being underweight in childhood, the leading risk factor for global loss of healthy life, occurred in severely underweight children who would be subject to clinical interventions (i.e., more than three standard deviations [SDs] from referent group median), the rest occurred in children only 1–3 SDs below the median. Even though the relative risks for the latter group are much lower, the number of children exposed to risk at this level is so great that the total attributable disease burden amounted to much more than that occurring in the severe category. Similarly, 52% of the attributable burden from physical inactivity occurred among those engaged in some but less than the recommended level of 2.5 h per week of moderate-intensity activity. For unsafe water, sanitation, and hygiene, almost all of the attributable disease burden was distributed among three of the five at-risk exposure categories, with approximately equal levels. This reflects the fact that the exposure categories were defined as the presence or absence of technology-based water and sanitation interventions. During decades of water and sanitation projects, many countries have “clustered” in a limited number of technology groups. There is likely to be large heterogeneity of exposure within each exposure category, however, because of factors such as quantity of water consumed and hygiene behavior [13].

**Table 4:**
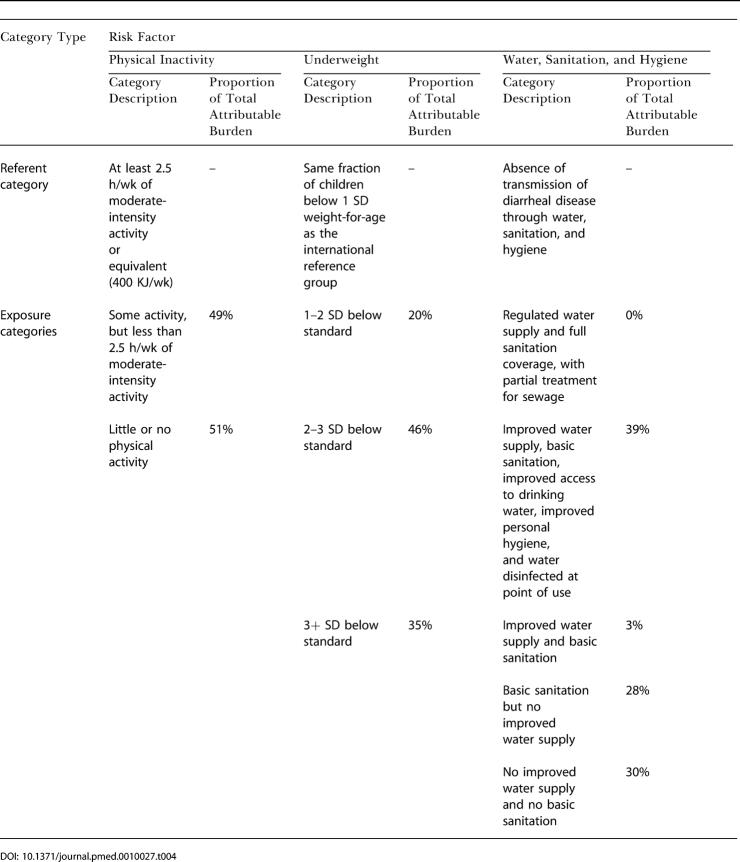
Distribution by Exposure Level of Attributable Burden Due to Selected Categorical Risk Factors

Figure 1 shows that at the aggregated level a substantial proportion of the attributable disease burden for high blood pressure, cholesterol, and BMI and low fruit and vegetable intake occurred in the “mid-range” exposures. For example, the second and third quartiles (i.e., half of attributable disease burden) occurred at the following levels: systolic blood pressure (SBP) of 130–150 mm Hg, cholesterol of 5.0–6.1 mmol/l, BMI of 25–32 kg/m^2^, and fruit and vegetable intake of 150–300 g/d (2–4 servings/d). This was similar to or greater than the amount of disease burden occuring among individuals with risk-factor levels above the commonly used, but arbitrary, thresholds for hypertension, hypercholesterolemia, and obesity indicated in Figure 1.

**Figure 2:**
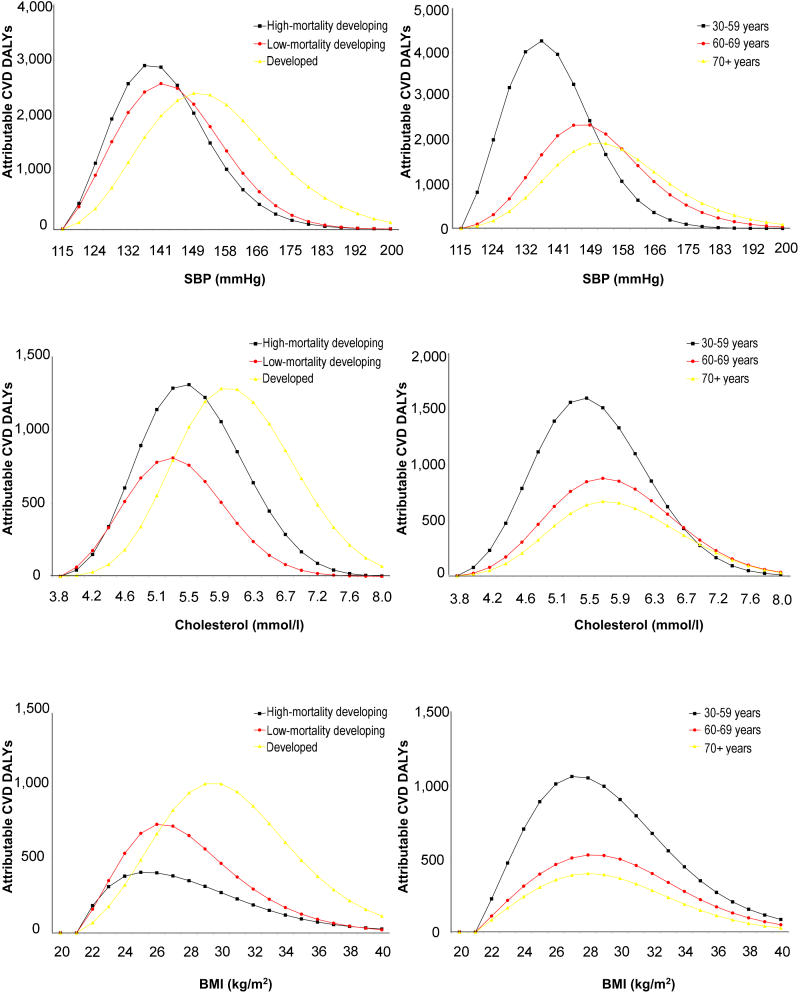
Distribution of Attributable Cardiovascular Disease Burden Due to BMI, Blood Pressure, and Cholesterol by Exposure Level, Age, and Level of Development Conventions as for Figure 1.

Despite the above finding on the important role of moderately elevated levels in total disease burden, the actual patterns of how disease burden is distributed among exposure levels varied across different regions and risk factors (Figure 2). For example, Figure 2 shows that a comparatively larger fraction of the disease burden attributable to elevated blood pressure, cholesterol, and BMI occurred at lower levels in developing regions compared to developed regions, mainly because of lower age-specific exposure levels in those populations. Figure 2 also shows that the skewness of the distribution of disease burden was not substantially different across different age groups for BMI. This is because the comparatively larger relative risk per unit BMI at younger ages (which leads to more right-hand skew) is counterbalanced by the comparatively lower BMI at younger ages (which leads to left-hand skew). Therefore, the different distributions of BMI-attributable disease burden by region resulted not from the different age structures of populations across major world regions, but rather from the lower *age-specific* BMI levels in those countries [14]. This is in contrast to blood pressure, for which disease burden in younger age groups occurred at lower exposures because the age patterns of exposure and relative risk do not entirely compensate.

## Discussion

The findings reported here should be considered within the context of limited available data and are subject to uncertainty, which varies across risk factors and is generally most marked in developing countries. Full discussion of uncertainty in the basic data sources and parameters is provided elsewhere and includes the uncertainties in estimates of disease incidence, duration, severity, and disability weighting [1,2]. Uncertainty in this risk assessment is by far dominated by absence or limitations of direct studies on exposure, hazard, and background disease burden, rather than parameter uncertainty, such as sampling error. This has motivated innovative assumptions and extrapolations even in the case of the best-studied risk factors in a limited number of countries [6]. While estimates of hazard size in individual studies were as much as possible adjusted for confounding effects, it is likely that residual confounding effects remain to some extent, hence leading to errors in estimation. Extrapolation of hazard from a limited number of studies to other populations is another source of potential error. While the robustness of relative measures of risk has been confirmed for more proximal factors in studies across populations [15,16,17], their extrapolation is an important source of uncertainty for more distal risks (e.g., childhood sexual abuse) or those whose effects are heterogeneous (e.g., alcohol and injuries versus alcohol and cancer). Direct exposure data for many risk factors are limited because of both difficulties in their measurement and underinvestment in risk-factor surveillance, especially in developing countries.

Of particular relevance to the current analysis is the fact that, due to data limitations, some risks were measured using categorical variables (e.g., indoor smoke from solid fuels, underweight, and physical inactivity) even though the health effects occur along a continuum. Other risk factors were represented using indirect or aggregate indicators (e.g., smoking impact ratio for accumulated hazards of smoking, and motor vehicle accident registries for alcohol-caused accidents) that do not allow quantification of risks along continua of exposure. Two important sources of uncertainty with implications for interventions are correlations among multiple risk factors and the skewness of risk-factor distribution. Because risks are often correlated (e.g., undernutrition, poor water and sanitation, and the use of solid fuels are more common among poor households in developing countries, and smokers are more likely to have poor diets), the contributions of high-exposure groups are likely to be underestimated. In addition to being a source of underestimation at higher exposure levels, risk-factor correlation implies that the same individuals and groups are at the high end of multiple risk factors. Positive (rightward) skewness of exposure distribution, not modeled here, would also lead to an underestimation of events occurring in those who were hypertensive, hypercholesterolemic, or obese. The importance of skewness is emphasized by the observation that the recent increase in the proportion of people who are overweight and obese (e.g., in the United States) has involved not only a shift in the distribution, but also increasing skewness, which has extended the high-exposure tail. Coupled with risk-factor correlation, this should motivate more systematic data collection and reporting to determine mean exposure as well as the complete shape of distribution.

The temporal nature of risk-factor exposure also has implications for the current cross-sectional estimates. There is an expectation that the size and rank order of risk-factor burden will alter in coming decades as a result of changes in exposure levels and delays between exposure and hazard. For example, it is predicted that by 2020 the leading risks to health will be (1) unsafe sex, principally because of HIV/AIDS and driven by increasing exposure, and (2) tobacco, because of market expansion of tobacco products in developing countries and delayed temporal effects on major health outcomes such as lung cancer [1].

This analysis in multiple age and exposure categories, or along a continuum of exposures, showed that, globally, a considerable proportion of the disease burden attributable to major risk factors occurred among those with only moderately raised levels, not the extremes such as those that define hypertension, obesity, or severe malnutrition. Further for many chronic-disease risk factors, such as tobacco and high blood pressure, as well as risk factors for injuries and sexual and reproductive health, significant proportions of disease burden occurred from events in middle ages, especially in developing countries. The concentration of disease burden from such a large number of risk factors in those below 60 years of age illustrates the large, and at times neglected, disease burden from risks that affect young adults in developing nations, with important consequences for economic development [18].

The distribution of risks and their determinants in a population have major implications for strategies of prevention. As stated by Rose, risk typically increases across the spectrum of a risk factor [8]. Rose's work led to one of the most fundamental axioms in disease prevention across risk factors: “A large number of people exposed to a small risk may generate many more cases than a small number exposed to high risk.” The analysis in this work showed that the risk factors for many of the major global diseases, such as lower respiratory infections, diarrhea, ischemic heart disease, and stroke exhibit such behavior, because they are caused by risks that occur along a continuum. Therefore, managing individual, high-risk crises, while appropriate for individuals, can only have a limited preventive effect at the population level and over long time periods because it relies on having adequate discriminative ability to predict future disease, and requires continued and expensive screening for new high-risk individuals. In contrast, population-based strategies that seek to shift the whole distribution of risk factors have the potential to substantially reduce total disease burden, and possibly over long time periods, if the interventions alter the underlying risk behaviors or their socioeconomic causes. This is particularly relevant in the context of risk factors such as those related to under- or overnutrition that affect societies in all stages of development. For example, a policy to reduce salt content in manufactured foods would result in a leftward shift in the population distribution of blood pressures and a surprisingly large reduction in cardiovascular disease [5]. Another example would be population-level measures that affected energy intake (such as the availability and price of energy-dense foods) and/or expenditure (such as the level of motorization and mechanization)—these can be expected to determine the distribution of a population's BMI levels, and hence largely determine its level of type 2 diabetes [1].

There were distinct patterns for the distribution of disease burden across risk factors, and across regions at various stages of development. At the extreme of these diverse patterns, for risk factors with acute exposure and acute outcomes, the underlying relationship is considerably more complex. For example while in many societies most alcohol-attributable injury (e.g., traffic accidents) involves people who on average drink moderate amounts [19], these people would be on the more extreme, high-risk spectrum in a different dimension: volume of drinking right before the injury. Therefore, if exposure to risk factors is clustered or the risk relationship does not follow a linear pattern, high-exposure groups may indeed play a disproportionately important role [20,21].

In summary, the analysis presented in this paper confirms and extends to a global level previous work indicating that the impact of many major risks is important across their exposure levels, not just among people with particularly high levels [8]. This analysis also illustrates that, beyond the general principle of population-wide shifts, there are important population-specific and risk-factor-specific patterns in how the burden of disease attributable to risk factors is distributed. Systematic assessment of multiple risks within any given population can provide the basis for selecting packages of interventions that include population-wide measures as well as highly targeted interventions provided to much smaller subsections of the population with constellations of major risks [1,5,18].

**Patient Summary**

**Background**

Health policy makers must understand existing health risks and which diseases cause the most health problems. The Global Burden of Disease database, maintained by the World Health Organization, collects information from around the world on risk factors such as malnutrition, childbirth, tobacco use, and cholesterol levels, as well as on diseases such as depression, blindness, and diarrhea. This information can be used to target health interventions.

**What Did the Researchers Do and Find?**

These researchers determined for 26 major risk factors the distribution of disease burden by age, sex, and exposure level. They found that many risk factors (such as high blood pressure and high cholesterol levels) occur across populations, and are not confined to one sex or age group. And for most risk factors, exposure to moderate risks (which is usually more common than exposure to severe risk) is responsible for causing most disease.

**What Do the Results Mean, and Who Will Use Them?**

Measures that reduce exposure to risk factors across whole populations, if only by a modest extent, can achieve large reductions in disease burden. This information is important for people who set global health policies and priorities.

**What Are the Problems with the Study?**

There are many challenges in estimating the impact of different major risks, each of which has distinct effects on a number of diseases. In addition, exposure to some risk factors today will cause disease only many years from now, so the picture will change over time. The major finding of the global distribution of many major risks is secure, but the exact extent of this remains uncertain due to the paucity of data in developing countries.

## Abbreviations

BMI: body mass index
DALY: disability-adjusted life-year
PAF: population attributable fraction
SBP: systolic blood pressure
SD: standard deviation
WHO: World Health Organization
